# Plasma metabolites as mediators in immune cell-pancreatic cancer risk: insights from Mendelian randomization

**DOI:** 10.3389/fimmu.2024.1402113

**Published:** 2024-06-12

**Authors:** Ke Zhang, Jie Zhu, Peng Wang, Yuan Chen, Zhengwang Wang, Xinyu Ge, Junqing Wu, Long Chen, Yipin Lu, Peng Xu, Jie Yao

**Affiliations:** ^1^ Dalian Medical University, Dalian, China; ^2^ Department of Hepatobiliary and Pancreatic Surgery, Northern Jiangsu People’s Hospital Affiliated Yangzhou University, Yangzhou, China

**Keywords:** pancreatic cancer, immune cells, Mendelian randomization, mediation analysis, plasma metabolites

## Abstract

**Background:**

Immune cells play a crucial role in the development and progression of pancreatic cancer, yet the causal relationship remains uncertain due to complex immune microenvironments and conflicting research findings. Mendelian randomization (MR), this study aims to delineate the causal relationships between immune cells and pancreatic cancer while identifying intermediary factors.

**Methods:**

The genome-wide association study (GWAS) data on immune cells, pancreatic cancer, and plasma metabolites are derived from public databases. In this investigation, inverse variance weighting (IVW) as the primary analytical approach to investigate the causal relationship between exposure and outcome. Furthermore, this study incorporates MR-Egger, simple mode, weighted median, and weighted mode as supplementary analytical approaches. To ensure the reliability of our findings, we further assessed horizontal pleiotropy and heterogeneity and evaluated the stability of MR results using the Leave-one-out method. In conclusion, this study employed mediation analysis to elucidate the potential mediating effects of plasma metabolites.

**Results:**

Our investigation revealed a causal relationship between immune cells and pancreatic cancer, highlighting the pivotal roles of CD11c+ monocytes (odds ratio, OR_IVW_=1.105; 95% confidence interval, 95%CI: 1.002–1.218; P=0.045), HLA DR+ CD4+ antigen-presenting cells (OR_IVW_=0.920; 95%CI: 0.873–0.968; P=0.001), and HLA DR+ CD8br T cells (OR_IVW_=1.058; 95%CI: 1.002–1.117; P=0.041) in pancreatic cancer progression. Further mediation analysis indicated that oxalate (proportion of mediation effect in total effect: -11.6%, 95% CI: -89.7%, 66.6%) and the mannose to trans-4-hydroxyproline ratio (-19.4, 95% CI: -136%, 96.8%) partially mediate the relationship between HLA DR+ CD8br T cells and pancreatic cancer in nature. In addition, our analysis indicates that adrenate (-8.39%, 95% CI: -18.3%, 1.54%) plays a partial mediating role in the association between CD11c+ monocyte and pancreatic cancer, while cortisone (-26.6%, 95% CI: 138%, -84.8%) acts as a partial mediator between HLA DR+ CD4+ AC and pancreatic cancer.

**Conclusion:**

This MR investigation provides evidence supporting the causal relationship between immune cell and pancreatic cancer, with plasma metabolites serving as mediators. Identifying immune cell phenotypes with potential causal effects on pancreatic cancer sheds light on its underlying mechanisms and suggests novel therapeutic targets.

## Introduction

As a frequently encountered malignant neoplasm, pancreatic cancer is characterized by its high malignancy and dismal prognosis (1). Over the last three decades, there has been a consistent rise in the incidence of pancreatic cancer. Projections indicate that by 2040, the overall incidence of pancreatic cancer is expected to surge by 30%. Pancreatic cancer typically presents as pancreatic adenocarcinoma, characterized by a grim prognosis, with an overall 5-year relative survival rate of approximately 10% (2, 3). In recent years, the ongoing development of immunotherapy has somewhat prolonged the overall survival of patients with advanced pancreatic cancer. Nevertheless, the prognosis remains challenging due to the intricacies of the immune microenvironment (4). Typically, risk factors associated with pancreatic cancer encompass unhealthy lifestyle behaviors, chronic pancreatitis, and a familial history of the disease (5, 6). As mounting evidence suggests, immune dysregulation and remodeling of the immune microenvironment emerge as risk factors contributing to the onset and progression of pancreatic cancer (7–9). Therefore, precise identification of immune-related risk factors and thorough exploration of their association with pancreatic cancer is paramount.

The immune microenvironment encompasses the complex milieu surrounding tumors, comprising cells, cytokines, extracellular matrix, blood vessels, and other factors. It exerts crucial regulatory influence over tumor growth and progression (10). As a complex regulatory network predominantly comprised of immune cells, the immune microenvironment demonstrates a dichotomous role in both anti-tumor and pro-tumor activities (11–13). While the immune system can effectively eradicate tumor cells via tumor immunosurveillance, tumors eventually circumvent immune surveillance by orchestrating an immunosuppressive microenvironment (14). This underscores the crucial need to precisely identify potent anti-tumor immune cells within the immune microenvironment and restore their cytotoxic effects on tumors. Such efforts could significantly bolster the response rates to immunotherapy and foster the development of novel immunotherapeutic strategies. Currently, there have been no randomized controlled trials (RCTs) investigating the relationship between immune cells and pancreatic cancer. Consequently, the potential causal relationships between specific immune cells and pancreatic cancer remain unknown.

Besides the intricate immune microenvironment, the distinctive metabolic traits of pancreatic cancer play a crucial role in fostering tumor cell survival, proliferation, and metastasis (15, 16). Previous studies have indicated that metabolic reprogramming in pancreatic cancer could induce changes in immune cell phenotypes, thereby fostering the establishment of suppressive immune microenvironments (17). Moreover, with the progress of metabolomics, an expanding array of plasma metabolites are being confirmed to play crucial roles in the early diagnosis of pancreatic cancer (18, 19). Previous studies have indicated that alterations in tumor cell metabolism are partially driven by the recruitment of immune cells (20). For instance, research by Pham et al. demonstrated that macrophages in the tumor microenvironment are reprogrammed into a pro-tumor (M2) phenotype through the uptake of lactate, a byproduct of tumor cell glycolysis. This uptake further activates HIF-1α, leading to increased expression of arginase 1 (Arg1) (21). Additionally, Geiger et al. found that high levels of Arg1 in the tumor microenvironment are associated with lower anti-tumor activity (22). Subsequently, a growing body of research has revealed that aberrant metabolites or intermediates of cancer metabolism play a crucial role in regulating the proliferation, differentiation, activation, and function of immune cells (23–25). For instance, in a study by Shane et al., elevated levels of tetrahydrobiopterin (BH4) were found to enhance the responses of CD4 and CD8 T cells, thereby amplifying their antitumor activity *in vivo*. Furthermore, BH4 administration in mice markedly reduced tumor growth and increased the population of intra-tumoral effector T cells (23). In a comprehensive metabolome-wide association study (MWAS), Zhong et al. systematically investigated the associations between genetically predicted blood metabolite levels and PDAC risk (26). Utilizing a two-sample MR approach, they identified 44 unique plasma metabolites that exhibit significant associations with PDAC risk (27). These findings indicate a potential causal relationship between plasma metabolites and the development, progression, and immune cell infiltration of tumors. Plasma metabolites may serve as intermediaries, mediating the causal association between immune cells and pancreatic cancer.

Despite the scarcity of RCTs evaluating the influence of plasma metabolites on immune cells and pancreatic cancer, it remains imperative to investigate the potential effects of plasma metabolites on both immune cells and pancreatic cancer.

MR provides a robust approach to investigate the causal relationship between exposure and outcomes by leveraging genetic variation, thereby mitigating confounding effects and reverse causation (28–30). Hence, it is apparent that the MR approach shares a conceptual resemblance with RCTs, yet it boasts broader applicability and superior cost-effectiveness in practical implementation. To probe the potential causal link between immune cells and pancreatic cancer, as well as the intermediary role of plasma metabolites, we devised a “two-step” MR analysis. This approach delves into the causal nexus between immune cells and pancreatic cancer, along with the mediating influence of plasma metabolites. Our research offers a fresh perspective on the mechanisms driving the onset and progression of pancreatic cancer, potentially paving the way for innovative metabolic intervention strategies.

## Materials and methods

### Research design

**Figure 1:**
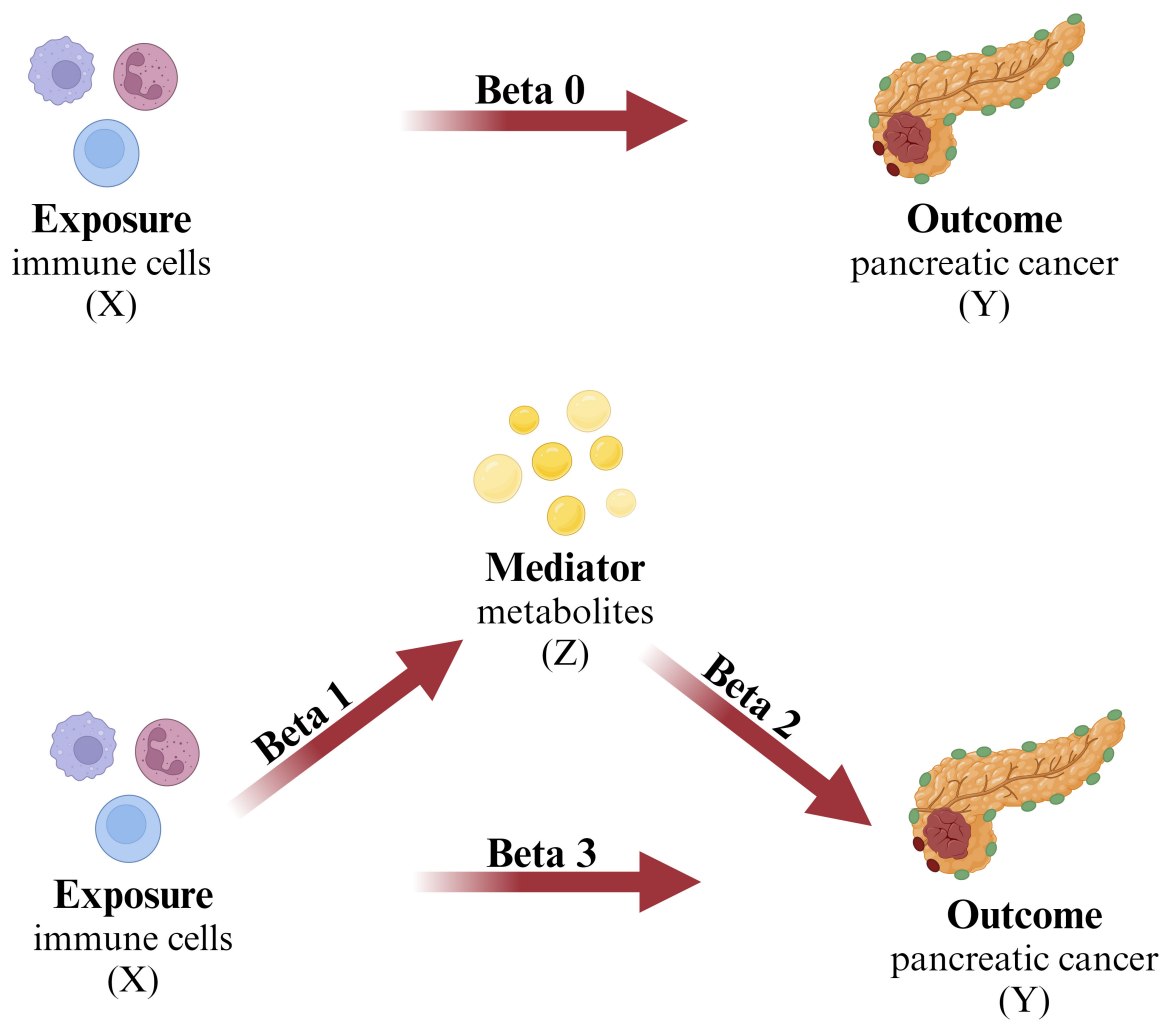
The mediation analysis X (exposure): immune cell; Y (outcome): pancreatic cancer; Z (mediator): plasma metabolites.

**Figure 2:**
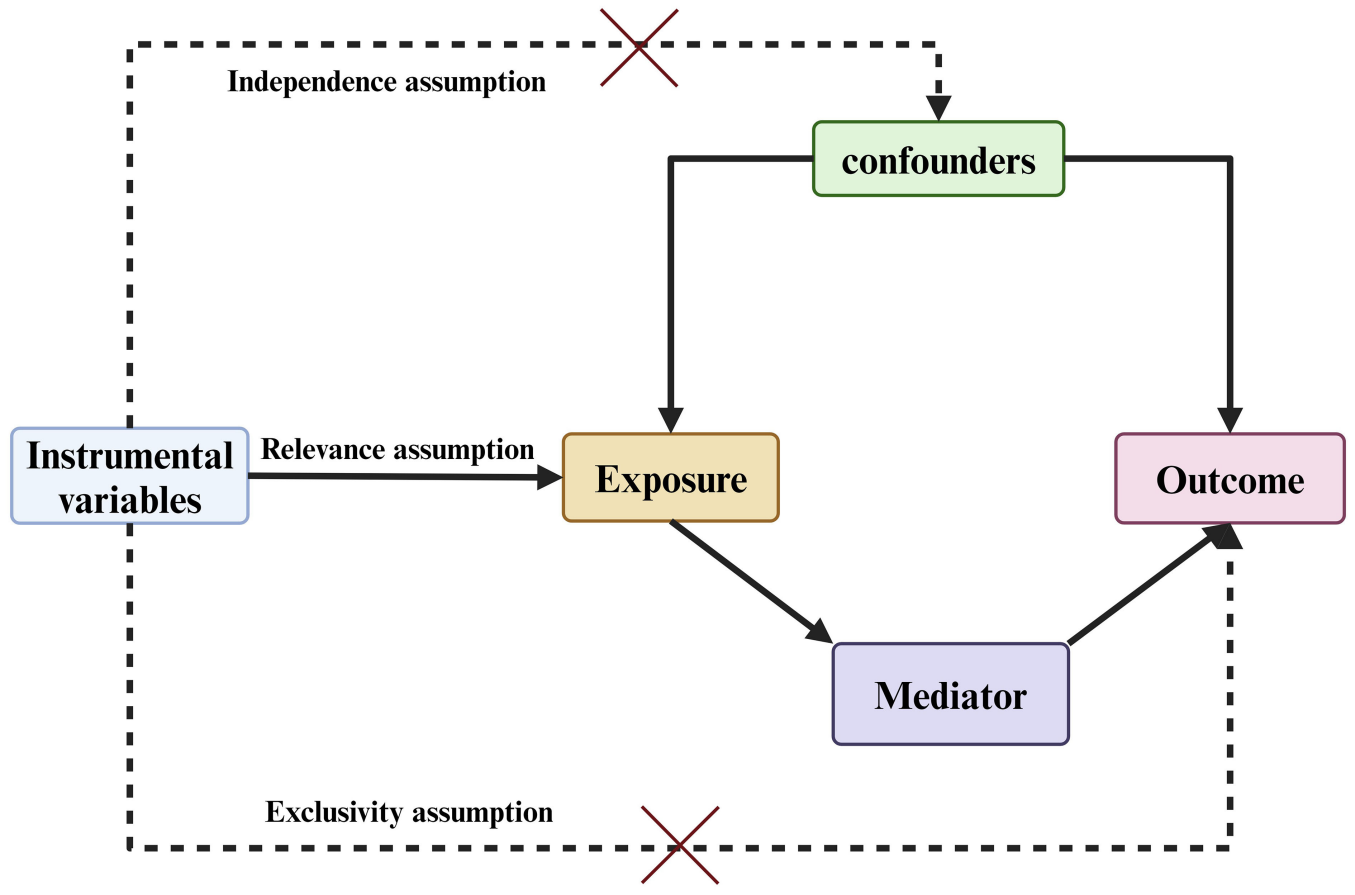
Overview of study design: Three Fundamental Assumptions of MR Analysis.

Based on bidirectional MR analysis using two independent samples and mediation analysis, this study investigated the causal relationship between 731 immune cell phenotypes and pancreatic cancer, and explored the potential modulation of this relationship by circulating metabolites. (**Figure 1**) At the outset, pancreatic cancer was designated as the primary outcome, while 731 immune cell phenotypes were identified as potential exposures, enabling an investigation into the comprehensive causal relationship between immune cell phenotypes and pancreatic cancer. Following this, we examined the respective proportions of causative links between 1400 plasma metabolites, acting as potential mediators, and the connections between pancreatic cancer and immune cell phenotypes. The instrumental variables (IV) employed in this investigation must satisfy the following criteria: 1. They must exhibit a strong correlation with the exposure factors. 2. The selected instrumental variables remain unaffected by confounding factors, and 3. They demonstrate a distinct relationship with the outcome event (29). (**Figure 2**) Furthermore, the GWAS data employed in this investigation were sourced from publicly available datasets and received approval from the respective institutional review boards in their studies.

### Source of data

#### The origin of the pancreatic cancer GWAS dataset

The statistical summary data for pancreatic cancer GWAS originates from the FinnGen consortium (https://www.finngen.fi/en), a nationwide longitudinal cohort study that encompasses genetic and electronic health record data. We incorporated GWAS data from 1626 pancreatic cancer patients, in accordance with the ICD-10 diagnostic criteria, sourced from the R10 version of FinnGen.

#### The GWAS data for immune cell phenotypes

The GWAS data on immune cell phenotypes were obtained from a cohort study involving 3,757 individuals of European ancestry (accession numbers from ebi-a-GCST0001391 to ebi-a-GCST0002121) (31). The research investigated the correlation between 731 immune cell phenotypes, covering B cells, dendritic cells, various stages of T cells, monocytes, NK cells, and regulatory T cells. These phenotypes included 118 absolute cell counts (AC), 389 median fluorescence intensities (MFI) reflecting surface antigen levels, 32 morphological parameters (MP), and 192 relative cell counts (RC), and their associations with 22 million single nucleotide polymorphisms (SNPs).

#### The GWAS data for plasma metabolites

The plasma metabolite GWAS dataset was obtained from the Canadian Longitudinal Study on Aging (CLSA), a cohort study involving 8299 participants (32). In this study, a total of 1458 metabolites were quantified with the Metabolon HD4 platform and were then batch-normalized. Only those with missing measurements in fewer than 50% of samples ( N=1091 ) were retained (32). Furthermore, the study utilized the Human Metabolome Database (HMDB) to identify 309 pairs of metabolites that share enzymes or transporters ( N=309 ) (32, 33). The ratio for each metabolite pair was then calculated by dividing the batch-normalized measurement value of one metabolite by the measurement value of the other metabolite within the same individual. Finally, a total of 1400 unique metabolites were evaluated in this study.

### Mendelian randomization analysis

#### IVs screening

The assumptions regarding IVs entail a substantial correlation with the exposure factor, while maintaining independence from the outcome variable. Moreover, only the exposure factor is presumed to have a direct impact on the outcome. Therefore, in order to screen for IVs associated with the exposure factor, we identified SNPs linked to the exposure factor, with a significance level set at $5\times 10^{-8}$ . Additionally, we further removed linkage disequilibrium (LD) effects for each SNP based on $\;\gamma^{2}<0.001$ and an interval of 10,000 kb (34, 35). Furthermore, to ensure the strong correlation between IVs and exposure factors, this study employed F-statistics to assess the strength of IVs. A causal inference was considered to have significant bias when the F-value was less than 10 (36). Finally, to control for the impact of confounding factors on instrumental variables, this study employed the Phenoscanner and R software packages, setting a $P\;<1\times 10^{-5}$ to eliminate palindromic SNPs and incompatible SNPs. This process excluded closely related to the occurrence of pancreatic cancer [BMI, smoking, drinking, diabetes, and pancreatitis, etc (37).] ensuring that the resulting instrumental variables were suitable for subsequent analysis.

#### MR analysis

To investigate the causal effects of exposure factors on outcome events and ensure robust results, we conducted a two-sample MR analysis using five different methods: IVW, Weighted Median, Simple Mode, Weighted Mode, and MR-Egger. The respective advantages and limitations of these methods are summarized in **Table 1**.

**Table 1:** Summary of five methods proposed for mendelian randomization.

Method	Consistency assumption	Advantages and limitations	Reference
IVW	All genetic variants are valid instruments, without pleiotropic effects	Most efficient (greatest statistical power), sensitive to pleiotropy	(38)
MR-Egger	InSIDE	Capable of detecting and addressing pleiotropy, albeit less precise, more susceptible to outliers, and lower in power than IVW	(39)
Weighted Median	Majority valid	Robust to outliers, sensitive to addition/removal of genetic variants	(40)
Weighted Mode	Plurality valid	Robust to outliers, sensitive to bandwidth parameter and addition/removal of genetic variants, generally conservative	(41)
Simple Mode	Plurality valid	Simple to compute and provides a quick check on the robustness of the results, less robust than Weighted Mode	(41)

InSIDE is the Instrument Strength Independent of Direct Effect assumption.

### The assessment of heterogeneity and horizontal pleiotropy

To maintain the validity of the independence and exclusivity assumptions, it is essential to ensure that IVs do not affect the outcome through factors unrelated to the exposure variable. Employing the MR-Egger intercept test to assess horizontal pleiotropy and ensure the robustness of the research findings. When the P-value of the MR-Egger intercept is less than 0.05, horizontal pleiotropy is deemed to be present; conversely, if the P-value is greater than 0.05, horizontal pleiotropy is considered to be absent (42). Employing Cochran’s Q statistic along with its corresponding P-value provides a quantitative evaluation of the heterogeneity among the selected IVs (43). In a final step, we conducted a sensitivity analysis using the “leave-one-out” approach to assess the impact of each SNP on the outcomes of the MR analysis (44). Statistical analysis and visualization of the results were conducted using the R software packages “TwoSample MR”, “Mendelian-Randomization”, and “ggplot2”.

### Analysis of the overall causal effect

To obtain the overall causal effect between immune cell phenotypes and pancreatic cancer, we executed a bidirectional two-sample MR analysis encompassing 731 immune cell phenotypes and their association with pancreatic cancer. All analyses were performed using R version 4.3.1 software (http://www.Rproject.org). In the absence of considering heterogeneity and horizontal pleiotropy, we employed the “Mendelian Randomization” R package to preliminarily evaluate the causal relationship between 731 immune cell phenotypes and pancreatic cancer using IVW (45). Moreover, this study employed MR-Egger regression analysis and weighted median-based analysis to further assess the reliability of the study findings (40, 46).

### Mediation analysis

Using a two-step MR approach, this study conducted mediation analysis to explore whether plasma metabolites mediate the causal pathway from immune cell phenotypes to pancreatic cancer outcomes. The overall effect of immune cell phenotypes on pancreatic cancer can be decomposed into the direct effect of immune cell phenotypes on pancreatic cancer and the indirect effect mediated by intermediates (47). In the first step of analysis, we computed the causal impact (Beta1) of immune cell phenotypes on the mediators. Following this, in the second step of analysis, we evaluated the causal impact (Beta2) of the mediators on pancreatic cancer. The proportion of mediated effect in the total effect was then obtained by calculating $\;R=\frac{Beta\;1\times Beta\;2}{Beta\;0}$ . The direct impact of the exposure on the outcome is represented as Beta 3=Beta 0−Beta 1×Beta 2 .

## Results

### Exploring the overall causal impact of immune cell phenotypes on pancreatic cancer

#### MR analysis

In order to investigate the overall causal impact of immune cell phenotypes on pancreatic cancer, we conducted two-sample MR analyses using five methods: IVW, weighted median, simple mode, weighted mode, and MR Egger. Subsequently, we employed a threshold of P<0.05 derived from the IVW method to detect 27 immune cell phenotypes causally associated with pancreatic cancer. Among these, 16 immune cell phenotypes, including CD11c+ monocyte % monocyte, are regarded as risk factors for pancreatic cancer (**Supplementary Figure 1**), while 11 immune cell phenotypes, such as CD4 Treg AC are regarded as protective factors for pancreatic cancer (**Supplementary Figure 2**). The summarized results of the MR analysis are provided in **Supplementary Tables 1**, **2**.

#### Analysis for horizontal pleiotropy and heterogeneity

To mitigate potential heterogeneity stemming from varying analysis platforms, experiments, populations, and other factors that may affect the results of MR analysis.

We evaluated heterogeneity using both the IVW and MR-Egger test methods, with P<0.05  indicating the presence of heterogeneity in the study (**Supplementary Table 4**). The results revealed that none of the intercepts from MR-Egger and IVW for the 27 immune cell phenotypes mentioned above were not statistically significant, indicating that the findings remained unaffected by any potential bias stemming from heterogeneity (**Supplementary Figure 3**). Furthermore, in order to mitigate potential confounding effects of IVs on outcomes via pathways beyond exposure, thereby safeguarding the assumptions of independence and exclusivity, we additionally employed the MR-Egger intercept method to assess the presence of horizontal pleiotropy in the data. The study findings indicate that the MR-Egger intercept for CX3CR1 on CD14- CD16- ( P=0.009772856 ) exhibited horizontal pleiotropy and was consequently excluded from subsequent analyses (**Supplementary Table 3**). The MR-Egger intercepts for the remaining 26 immune cell phenotypes ( P>0.05 ) were not statistically significant, suggesting an absence of genetic pleiotropy bias. Through leave-one-out sensitivity analysis, the study results were further validated, revealing no indication of SNPs impacting the overall causal relationship (**Supplementary Figure 4**).

### Identifying immune cell phenotypes associated with pancreatic cancer

Based on the study findings, in order to identify immune cell phenotypes associated with pancreatic cancer for further analysis, we initially screened for immune cell phenotypes showing consistent directional effects ( OR〈1 or OR〉1 ) across five different MR analysis methods. This screening process resulted in the identification of 22 immune cell phenotypes (**Supplementary Figure 5**). Subsequently, we conducted reverse MR analysis on the 22 immune cell phenotypes in relation to pancreatic cancer, excluding those with reverse causal effects (**Supplementary Table 5**). Ultimately, we identified 20 immune cell phenotypes associated with pancreatic cancer for further mediation analysis.

### Mediating effects of plasma metabolites on immune cell−pancreatic cancer risk

To investigate the mediating effect of plasma metabolites on the association between immune cell and pancreatic cancer, with the aim of elucidating the underlying mechanisms by which immune cells influence pancreatic cancer. Based on two-step MR analysis, we first performed two-sample MR analysis on 1400 plasma metabolites with pancreatic cancer and then, plasma metabolites with consistent OR values were selected as pancreatic cancer-associated plasma metabolites for further analysis (**Supplementary Table 6**).

**Figure 3:**
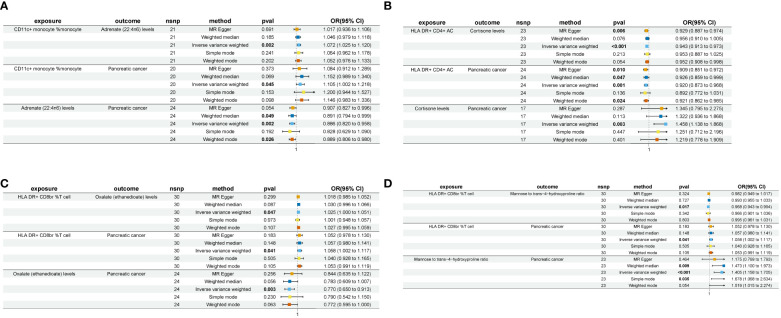
A forest plot of the four sets of plasma metabolites exhibiting “immune cell phenotype - pancreatic cancer” mediating effects nsnp, number of single nucleotide polymorphism; pval, p-value; OR, odds ratio. **(A)** CD11c+ monocyte % monocyte-Adrenate (22:4n6)-Pancreatic cancer. **(B)** HLA DR+ CD4+ AC-Cortisone-Pancreatic cancer. **(C)** HLA DR+ CD8br %T cell-Oxalate (ethanedioate)-Pancreatic cancer. **(D)** HLA DR+ CD8br %T cell-Mannose to trans-4-hydroxyproline ratio-Pancreatic cancer.

In our investigation, we first examined the mediating effect (Beta1) of immune cell phenotype on plasma metabolites (**Supplementary Table 7**). Subsequently, employing the same methodology, we analyzed the mediating effect (Beta2) of plasma metabolites on pancreatic cancer (**Supplementary Table 8**). Ultimately, our analysis identified four pairs of plasma metabolites that serve as mediators in the relationship between immune cell phenotype and pancreatic cancer (**Figure 3**).

The levels of adrenate (22:4n6) were found to negatively modulate pancreatic cancer concerning CD11c+ monocyte %monocyte (Mediated effect, ME=-0.00837; Mediated proportion, MP=-8.39%) (**Table 2**). Oxalate (ethanedioate) levels exhibited a negative regulatory effect on pancreatic cancer with respect to HLA DR+ CD8br %T cell (ME=-0.00655; MP=-11.6%) (**Table 2**). Cortisone levels negatively regulated pancreatic cancer in conjunction with HLA DR+ CD4+ AC (ME=-0.0223; MP=-26.6%) (**Table 2**). Lastly, the mannose to trans-4-hydroxyproline ratio negatively influenced pancreatic cancer in association with HLA DR+ CD8br %T cell (ME=-0.011; MP=-19.4%) (**Table 2**).

**Table 2:** Four pairs of plasma metabolites with “immune cell phenotype - pancreatic cancer” mediating effects.

Immune cell	Metabolite	Outcome	Mediated effect	Mediated proportion
CD11c+ monocyte %monocyte	Adrenate (22:4n6) levels	Pancreatic cancer	-0.00837 (-0.0183, 0.00153)	-8.39% (-18.3%, 1.54%)
HLA DR+ CD4+ AC	Cortisone levels	Pancreatic cancer	-0.0223 (-0.116, 0.0712)	-26.6% (138%, -84.8%)
HLA DR+ CD8br % T cell	Oxalate (ethanedioate) levels	Pancreatic cancer	-0.00655 (-0.0508, 0.0377)	-11.6% (-89.7%, 66.6%)
HLA DR+ CD8br % T cell	Mannose to trans-4-hydroxyproline ratio	Pancreatic cancer	-0.011 (-0.0768, 0.0548)	-19.4% (-136%, 96.8%)

## Discussion

Pancreatic cancer, characterized by its high malignancy, aggressive biological behavior, and poor prognosis, presents a persistent threat to global health (2, 48). Immune cells play a crucial role in the initiation and progression of pancreatic cancer (49). Despite the growing interest in immune cells and immunotherapy in pancreatic cancer research, the intricate dynamics between immune cells and pancreatic cancer remain largely unexplored. Metabolic reprogramming, as a pivotal biological phenomenon in tumor tissue, exerts a profound influence on tumor cell growth, proliferation, and survival (50).

Emerging evidence suggests that intricate metabolic alterations within the tumor microenvironment activate immune cells via diverse intrinsic or extrinsic pathways. These complex interactions are pivotal in driving multiple phenotypes such as tumor proliferation, migration, and drug resistance (51–53). Therefore, the reciprocal interplay between immune cells and metabolism emerges as an intriguing topic warranting in-depth exploration. Nevertheless, due to the relatively small number of pancreatic cancer samples available, acquiring high-quality tissue specimens remains a formidable challenge. Furthermore, the complexity of metabolic products *in vivo* imposes limitations on the depth and breadth of research endeavors. Moreover, the absence of standardized criteria for investigating immune cell phenotypes has contributed to challenges in comparing and verifying findings. Therefore, in order to clarify whether immune cells impact the development of pancreatic cancer by mediating endogenous metabolites, we employed a two-step MR analysis to investigate whether plasma metabolites act as mediating factors affecting the causal relationship between immune cells and pancreatic cancer. The outcomes of our investigation reveal a causal relationship between immune cells and pancreatic cancer. Furthermore, mediation analysis provides additional evidence supporting the mediating effect of plasma metabolites in the causal relationship between immune cells and pancreatic cancer. This discovery may provide fresh insights into the mechanisms driving the onset and progression of pancreatic cancer, thereby offering novel intervention targets for therapeutic strategies.

Adrenate (22:4n6) is found to negatively mediate the causal relationship between CD11c+ monocyte and pancreatic cancer (ME=-0.00837, MP=-8.39%) (**Table 2**). Recent investigations have revealed the significant involvement of CD11c+ monocytes in the early metastatic process of pancreatic cancer (54), aligning with our own research findings (OR_IVW_=1.105, 95%CI=1.002–1.218) (**Figure 3A**). Adrenate (22:4n6), a 22-carbon, 4-double-bond ω-6 fatty acid also known as adrenic acid, plays a crucial biological role in the human body. It contributes to various essential functions, including the structural and functional integrity of cellular membranes, cell signaling processes, and the regulation of inflammation (55). Research findings indicate that its peroxidation plays a critical role in regulating the sensitivity of various drug-resistant tumor cells to ferroptosis (56, 57). The observations indicate that adrenate (22:4n6) exhibits anti-tumor effects, consistent with the outcomes of our study (OR_IVW_=0.886, 95%CI=0.820–0.958) (**Figure 3A**). In a study of murine peritonitis, adrenate (22:4n6) was found to suppress leukotriene B4 (LBT4) synthesis in neutrophils and promote their apoptosis, thereby facilitating phagocytosis by monocyte-derived macrophages and ultimately exerting an anti-inflammatory effect (58). The findings above suggest that adrenate (22:4n6) may act as a mediator, mitigating local inflammatory responses within the tumor microenvironment by inhibiting leukotriene synthesis in immune cells, thereby modulating immune cell effects on pancreatic cancer.

HLA-DR, a key component of the major histocompatibility complex (MHC) class II molecules, is commonly found on specialized antigen-presenting cells. It binds external peptides and delivers them to antigen-specific CD4+ helper (Th) T lymphocytes (59). Research findings suggest that HLA-DR+ CD4+ T lymphocytes play a critical role as activated T cells in anti-tumor immune responses (60). They regulate and enhance the activities of other immune cells while also directly targeting tumor cells for cytotoxic effects (61). Our study supports this viewpoint, suggesting an inverse correlation between HLA-DR+ CD4+ antigen-presenting cells and pancreatic cancer (OR_IVW_=0.920, 95%CI=0.873–0.968) (**Figure 3B**). Further mediation analysis revealed that cortisone exerts a negative mediating effect (ME=-0.0223, MP=-26.6%) (**Table 2**). Cortisone, a natural glucocorticoid present in the human body, plays a critical role in maintaining metabolic balance, regulating cell growth, and modulating inflammatory responses (62). Research has uncovered that cortisone binding to glucocorticoid receptors (GR) in pancreatic cancer elicits tumor proliferation, metastasis, and gemcitabine resistance via diverse signaling cascades (63). Cortisol, the most representative hormone among glucocorticoids, is commonly used to treat inflammatory diseases. Traditionally, exogenous glucocorticoids (GCs) have been widely recognized for their immunosuppressive effects (62). However, recent studies on endogenous GCs have revealed that they can both promote and inhibit T cell immunity (64). For instance, current research indicates that endogenous GCs induce differential T cell differentiation to maintain a balance, thereby preventing excessive immune responses (65). The results reported in the previous study are consistent with our own findings (OR_IVW_=1.458, 95%CI=1.138–1.868) (**Figure 3B**), indicating that cortisone acts as a mediator, attenuating the impact of HLA DR+ CD4+ T lymphocytes on pancreatic cancer.

HLA DR+ CD8br T cells are commonly acknowledged as activated cytotoxic T lymphocytes (CTLs), with the conventional viewpoint asserting their significance as a key element in cellular immunity, playing a crucial role in the antitumor process (66). Nevertheless, recent investigations have revealed that excessive activation of CTLs could foster the development of an immunosuppressive microenvironment. In Yang et al.’s investigation, it was observed that in lung adenocarcinoma, hyperactivated CTLs induce tumor cells to produce prostaglandin E2 (PGE2) and recruit myeloid-derived suppressor cells (MDSCs) via the Fas/FasL signaling axis, thereby fostering tumor immune evasion (67). The above viewpoint is consistent with the findings of this study (OR_IVW_=1.058, 95%CI=1.002–1.117) (**Figures 3C**, **3D**). Further mediation analysis revealed the participation of two plasma metabolites in mediating the causal effect of HLA DR+ CD8br T cells on pancreatic cancer. Intracellular accumulation of oxalate, a passive metabolite synthesized by various cells (68), has been found to activate nicotinamide adenine dinucleotide phosphate (NADPH) oxidase, thereby inducing reactive oxygen species (ROS)-mediated oxidative stress (OS) and subsequent tissue damage (69, 70). In light of OS emerging as a pivotal biological phenomenon in tumor progression, the dual therapeutic strategy aimed at modulating the redox status (i.e., pro-oxidant therapy and/or antioxidant therapy) is gaining increasing attention (71, 72). OS acts as a double-edged sword in tumor biology. On one hand, it promotes malignant behaviors in tumors, while on the other hand, it triggers cytotoxic OS within tumor cells, which can be exploited for therapeutic purposes (73, 74). For instance, commonly used chemotherapy drugs like doxorubicin, cisplatin, and 5-fluorouracil can either directly or indirectly enhance the accumulation of ROS, leading to tumor cell death (75, 76). A cohort study on dialysis patients revealed a significant elevation in IL-16 levels, which correlated with plasma oxalate concentrations (77). Similarly, research on colorectal cancer indicated that high IL-16 levels inhibited the cytotoxicity of CD8+ T cells in the immune microenvironment (78). These findings suggest that plasma oxalate may impair CD8+ T cell cytotoxicity through the upregulation of cytokines like IL-16.

In summary, oxalate might trigger apoptosis in tumor cells through OS, consequently attenuating the positive causal relationship between HLA DR+ CD8br T cells and pancreatic cancer.

Intracellular uptake of mannose, a natural C-2 epimer of glucose, occurs via facilitated diffusion through glucose transporters (GLUTs) situated on the cell membrane (79). Subsequently, within the cell, mannose undergoes enzymatic conversion catalyzed by phosphomannose isomerase (MPI) to yield fructose-6-phosphate, thereby entering the glycolytic pathway (80). However, the limited endogenous levels of mannose result in minimal energy-providing capacity. In recent years, research has shown that the combination of mannose with conventional chemotherapy impacts the levels of Bcl-2 family anti-apoptotic proteins, resulting in increased sensitivity of cells to chemotherapy drugs (81). In addition to its direct anti-tumor effects, mannose also possesses immunomodulatory properties. In a study conducted by Zhang et al., it was found that increasing the dosage of mannose in the drinking water of mice suppressed immune cell infiltration in autoimmune diabetes and airway inflammation, while enhancing the infiltration of regulatory T cells (Tregs) (82). Further *in vitro* experiments confirmed that mannose stimulates the differentiation of Tregs in both humans and mice by promoting the activation of TGF-β (82, 83). In a separate investigation focusing on mannose, it was revealed that supra-physiological concentrations of mannose can inhibit macrophage activation (84). From this perspective, mannose not only exerts direct anti-tumor effects but also demonstrates negative immunomodulatory effects.

Hydroxyproline, an essential structural amino acid in the body, plays a crucial role in collagen synthesis. Studies have indicated that an increase in hydroxyproline concentration in the blood promotes the distant metastasis of pancreatic cancer (85). Moreover, studies have demonstrated that hydroxyproline enhances IFN-γ levels, thereby promoting the expression of PD-L1 and inhibiting tumor autophagy (86).

The specific role of trans-4-hydroxyproline, a variant of hydroxyproline, in cancer pathogenesis remains largely elusive. A large-scale prospective study on plasma metabolites in prostate cancer identified a significant association between elevated plasma levels of trans-4-hydroxyproline and heightened risk of prostate cancer (87). In summary, further investigations are needed to fully elucidate the specific roles of mannose and trans-4-hydroxyproline in tumorigenesis. Our study highlights the mannose to trans-4-hydroxyproline ratio as a risk factor, exerting a negative regulatory effect on HLA DR+ CD8br % T cell responses in pancreatic cancer (ME=-0.011, MP=-19.4%) (**Table 2**).

One of the major strengths of our study lies in the application of MR, which employs SNPs as IVs to analyze the relationship between exposure and outcome. To our knowledge, this study represents the first attempt to employ MR analysis using the most recent and comprehensive GWAS data to investigate the causal relationship between immune cells and pancreatic cancer. Compared to RCTs, MR helps mitigate confounding factors and prevents interference from reverse causality. We employ two-steps MR to investigate the linear relationship between exposure and outcome, and employ mediation analysis to explore potential non-linear associations of plasma metabolites. Furthermore, the utilization of data samples derived from the identical population minimizes biases originating from population heterogeneity. Conducting multiple sensitivity analyses enhances the resilience and dependability of the outcomes. Nevertheless, our study still faces several noteworthy limitations. Firstly, the bulk of statistical data in GWAS originates from individuals of European descent, limiting the generalizability of our findings to diverse ethnic populations. Consequently, additional research is needed to extend our results to other racial groups. Secondly, regarding the selection of outcome GWAS data, this study utilized GWAS data from FinnGen. Although the FinnGen dataset is smaller in scale compared to the larger GWAS datasets from the Pancreatic Cancer Cohort Consortium (PanScan) and the Pancreatic Cancer Case-Control Consortium (PanC4), FinnGen, which commenced in the fall of 2017, offers an advantage in terms of data timeliness (88). Additionally, Zhong et al. have already analyzed the association of plasma metabolites with pancreatic cancer using PanScan and PanC4 data (26, 27). Our study aims to explore different GWAS datasets to identify new potential mediators, thereby providing complementary insights and enhancing the robustness of our findings. Thirdly, the causal relationship between immune cells and pancreatic cancer is influenced by numerous factors, and there may still be unidentified confounding variables between exposure and outcome, which could introduce bias into our results.

## Conclusion

In summary, this study presents genetic evidence of the causal relationship between immune cells and pancreatic cancer through MR analysis. In our investigation, we found that higher levels of HLA-DR+ CD4+ T lymphocytes were associated with a decreased risk of pancreatic cancer. Notably, cortisone was found to significantly modulate this effect, explaining 26.6% of the association. Furthermore, the investigation revealed a direct association between HLA DR+ CD8 T cells and pancreatic cancer. Notably, the mannose to trans-4-hydroxyproline ratio emerged as a key mediator, explaining 19.4% of the total effect. Additionally, oxalate showed significant mediation, accounting for 11.6% of the total effect. In essence, higher levels of HLA DR+ CD8 T cells corresponded to an increased risk of pancreatic cancer, with the mannose to trans-4-hydroxyproline ratio and oxalate serving as intermediaries. In addition, the findings revealed a positive correlation between CD11c+ monocytes and pancreatic cancer incidence, with adrenate serving as a mediator, accounting for 8.39% of the effect. In conclusion, the findings from the above study may offer fresh insights into the mechanisms underlying the initiation and advancement of pancreatic cancer, thus presenting novel metabolic intervention targets for therapeutic strategies.

## Data availability statement

Publicly available datasets were analyzed in this study. This data can be found here: https://www.finngen.fi/en.

## Ethics statement

Ethical approval was deemed unnecessary for the human studies as the original studies had undergone ethical review and approval, as documented in their respective publications. Informed consent was obtained from all participants involved in the original genome-wide association studies. The studies were conducted in accordance with local legislation and institutional requirements. Participants provided written informed consent to participate in this study.

## Author contributions

KZ: Writing – original draft, Writing – review & editing. JZ: Conceptualization, Data curation, Investigation, Validation, Writing – review & editing. PW: Writing – review & editing. YC: Writing – review & editing. ZW: Writing – review & editing. XG: Writing – review & editing. JW: Writing – review & editing. LC: Writing – review & editing. YL: Writing – review & editing. PX: Conceptualization, Methodology, Writing – review & editing. JY: Writing – review & editing.
