# Supplementary material for: Plasma metabolites as mediators in immune cell-pancreatic cancer risk: insights from Mendelian randomization
Source: Front Immunol. 2024 Jun 12;15:1402113. doi: 10.3389/fimmu.2024.1402113 (PMC11199692; doi:10.3389/fimmu.2024.1402113)
Supplement: Supplementary file 1 [file DataSheet_1.docx]

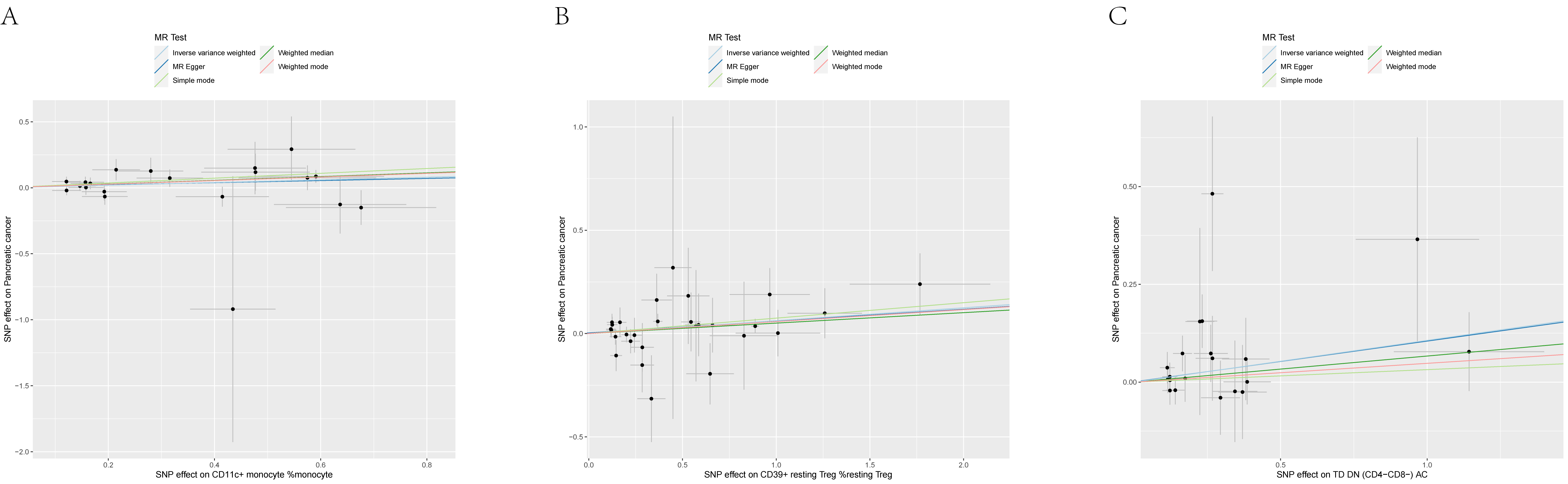


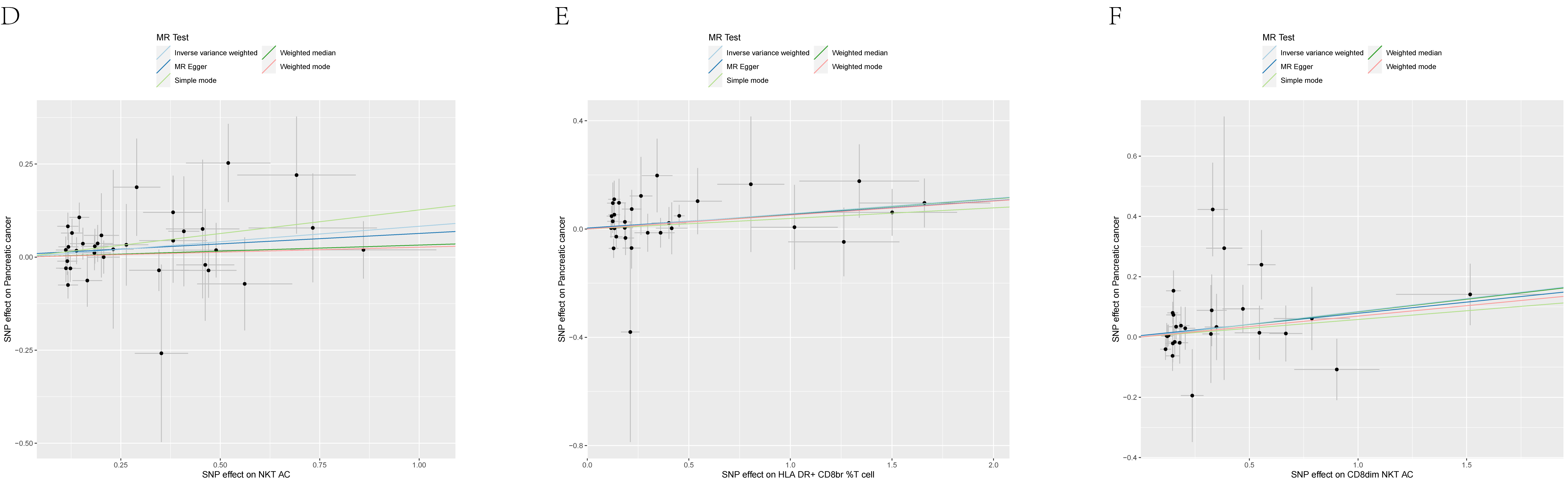


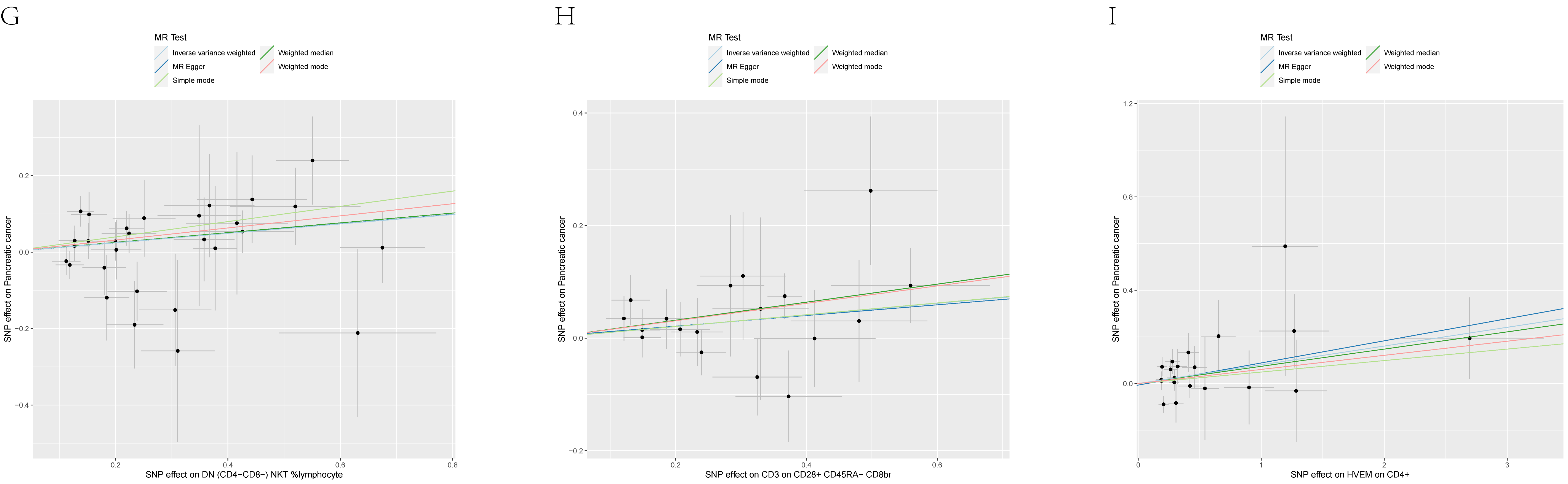


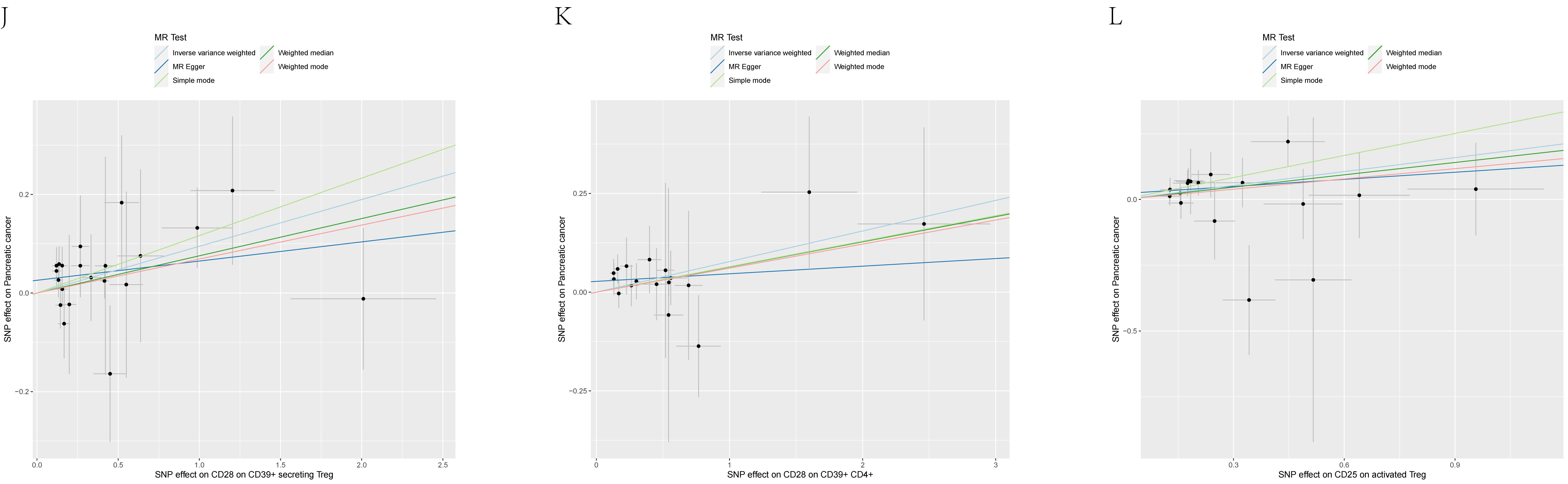


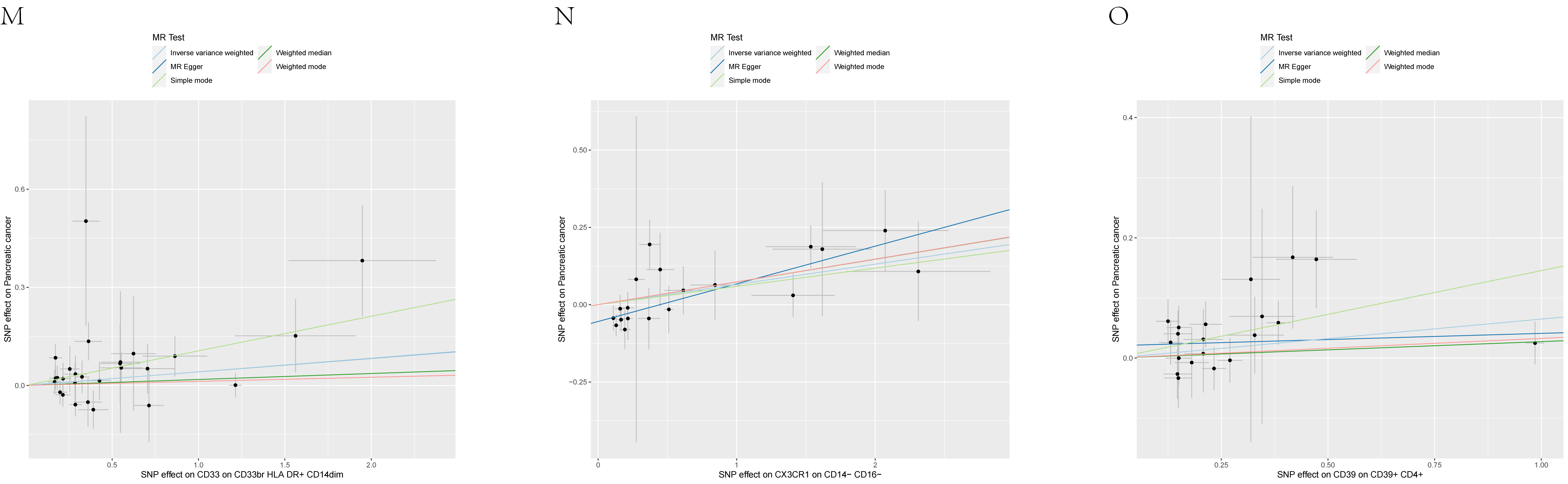


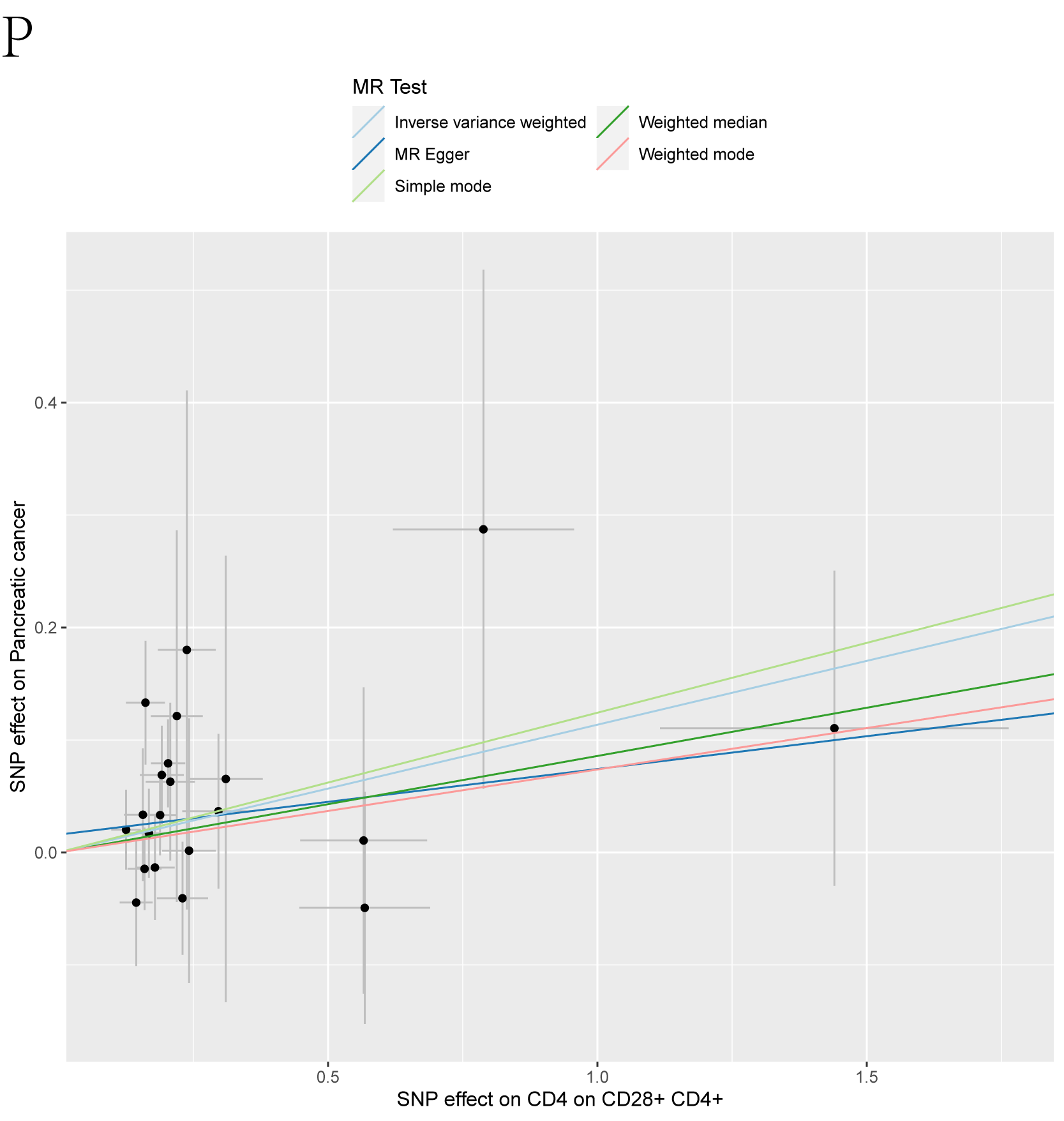


Supplementary Fig. 1 The immunophenotypes of 16 immune cells, considered as risk factors for pancreatic cancer, were assessed based on MR analysis results from five different approaches, with a significance threshold of Ρ<0.05.

(A) CD11c+ monocyte %monocyte，(B) CD39+ resting Treg %resting Treg，(C) TD DN (CD4-CD8-) AC, (D) NKT AC, (E) HLA DR+ CD8br %T cell, (F) CD8dim NKT AC, (G) DN (CD4-CD8-) NKT %lymphocyte, (H) CD3 on CD28+ CD45RA- CD8br, (I) HVEM on CD4+, (J) CD28 on CD39+ secreting Treg, (K) CD28 on CD39+ CD4+, (L) CD25 on activated Treg, (M) CD33 on CD33br HLA DR+ CD14dim, (N)CX3CR1 on CD14- CD16-, (O) CD39 on CD39+ CD4+, (P)CD4 on CD28+ CD4+。IVW: inverse variance weighting
