# Supplementary material for: Plasma metabolites as mediators in immune cell-pancreatic cancer risk: insights from Mendelian randomization
Source: Front Immunol. 2024 Jun 12;15:1402113. doi: 10.3389/fimmu.2024.1402113 (PMC11199692; doi:10.3389/fimmu.2024.1402113)
Supplement: Supplementary file 2 [file DataSheet_2.docx]

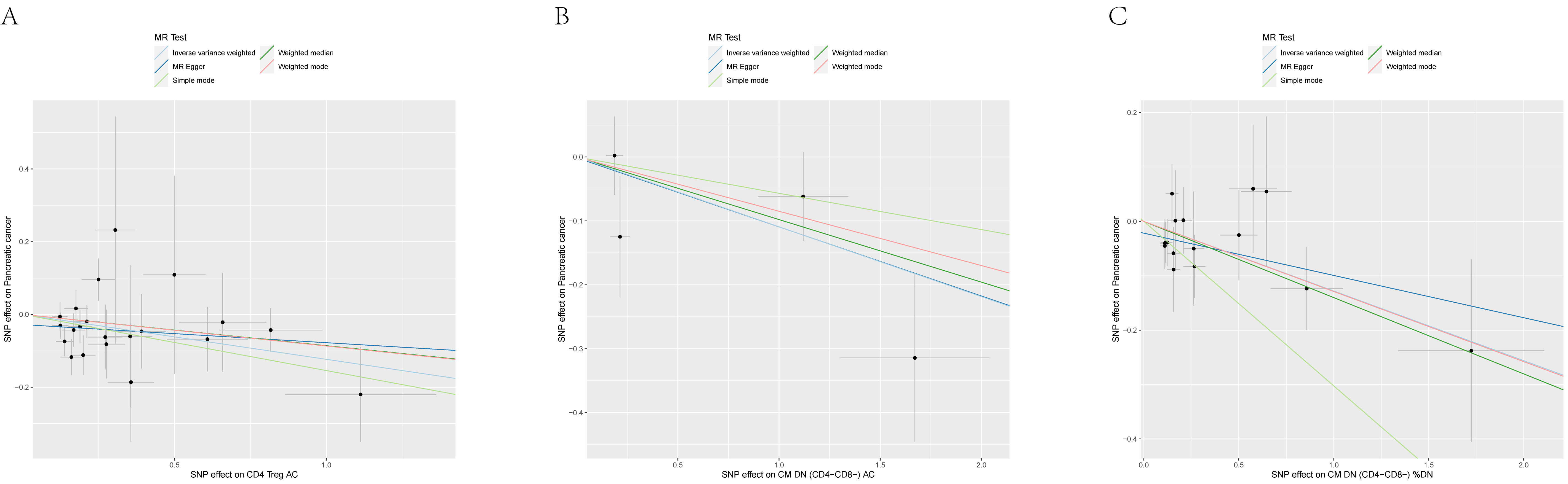


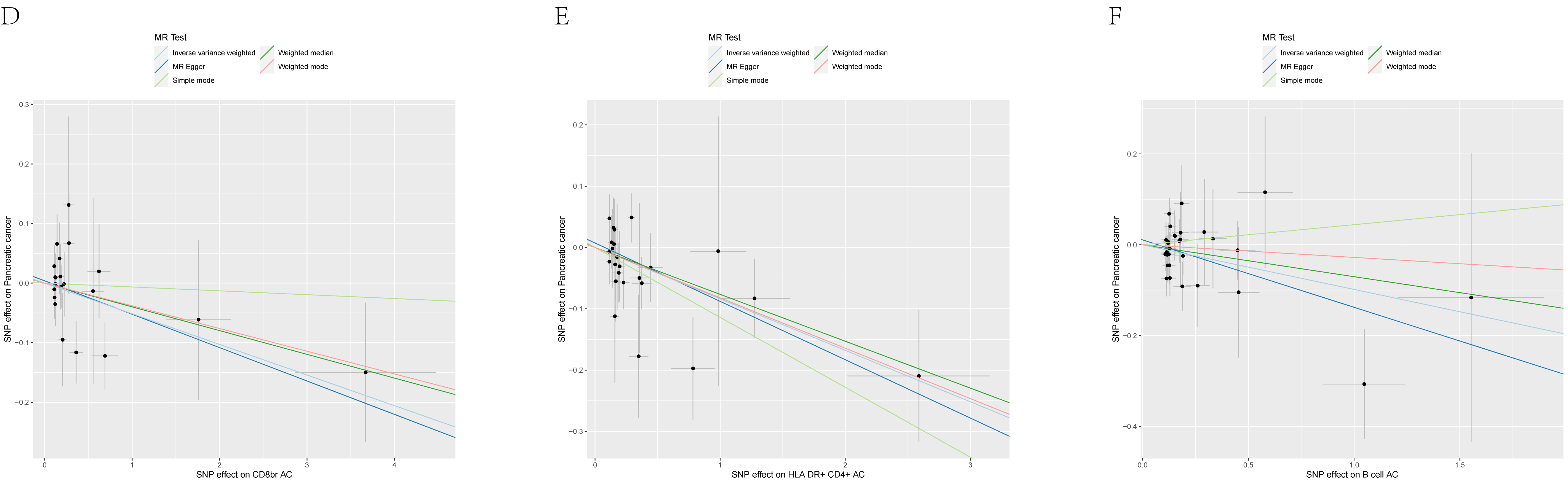


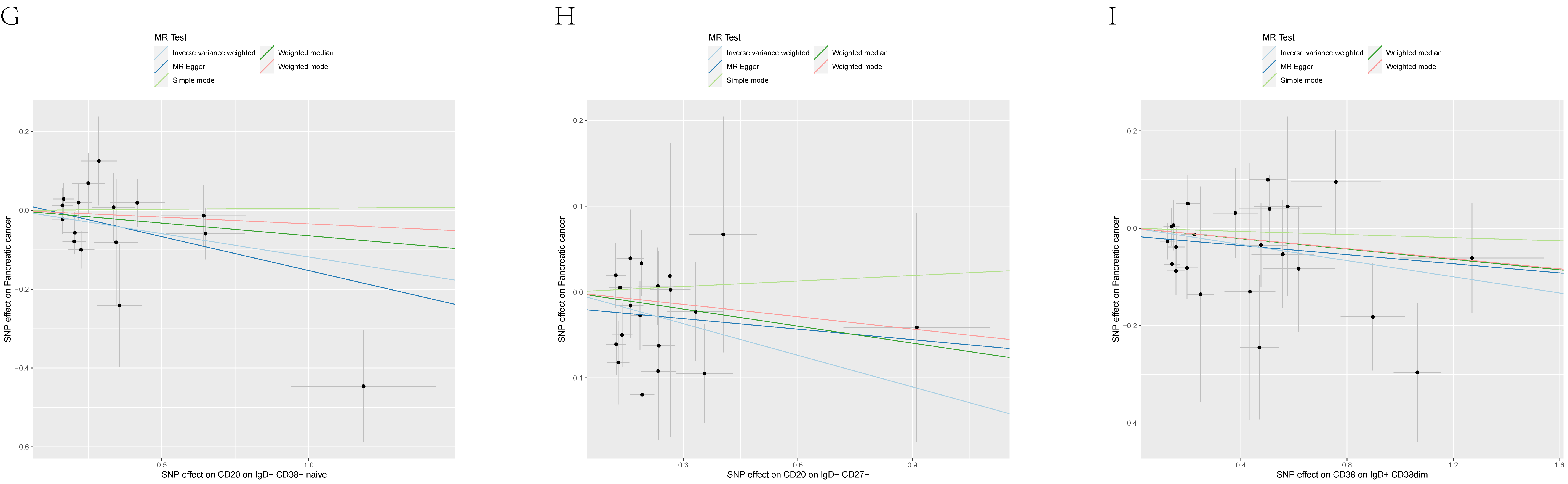


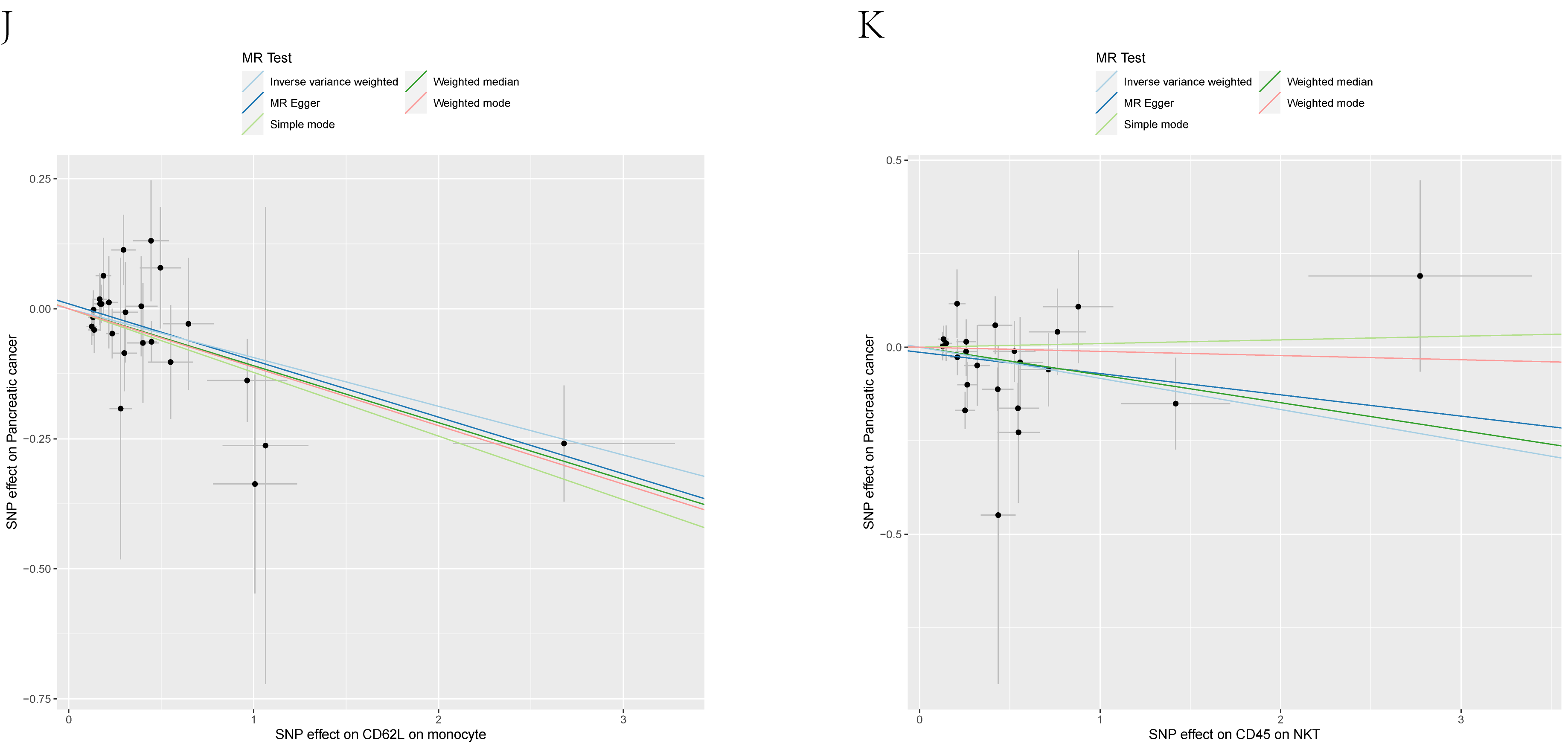


Supplementary Fig. 2 The immunophenotypes of 11 immune cells, considered as protective factors for pancreatic cancer, were assessed based on MR analysis results from five different approaches, with a significance threshold of Ρ<0.05.

(A) CD4 Treg AC，(B) CM DN (CD4-CD8-) AC，(C) CM DN (CD4-CD8-) %DN, (D) CD8br AC, (E) HLA DR+ CD4+ AC, (F) B cell AC, (G) CD20 on IgD+ CD38- naive, (H) CD20 on IgD- CD27-, (I) CD38 on IgD+ CD38dim, (J) CD62L on monocyte, (K) CD45 on NKT.
