# Supplementary material for: Plasma metabolites as mediators in immune cell-pancreatic cancer risk: insights from Mendelian randomization
Source: Front Immunol. 2024 Jun 12;15:1402113. doi: 10.3389/fimmu.2024.1402113 (PMC11199692; doi:10.3389/fimmu.2024.1402113)
Supplement: Supplementary file 3 [file DataSheet_3.docx]

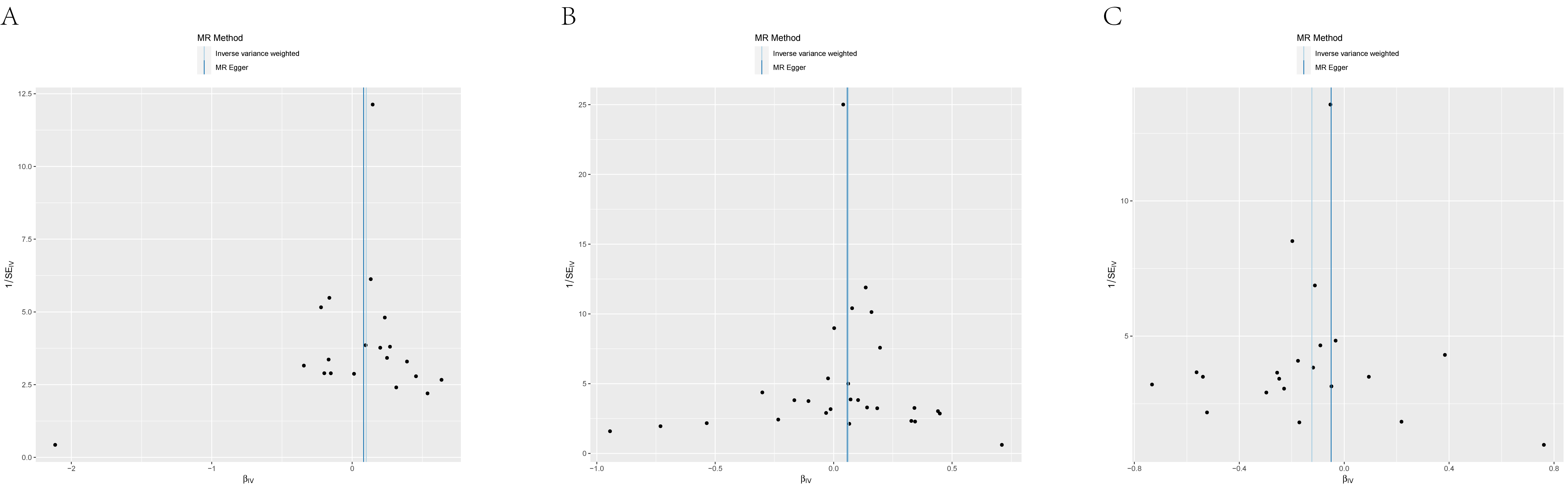


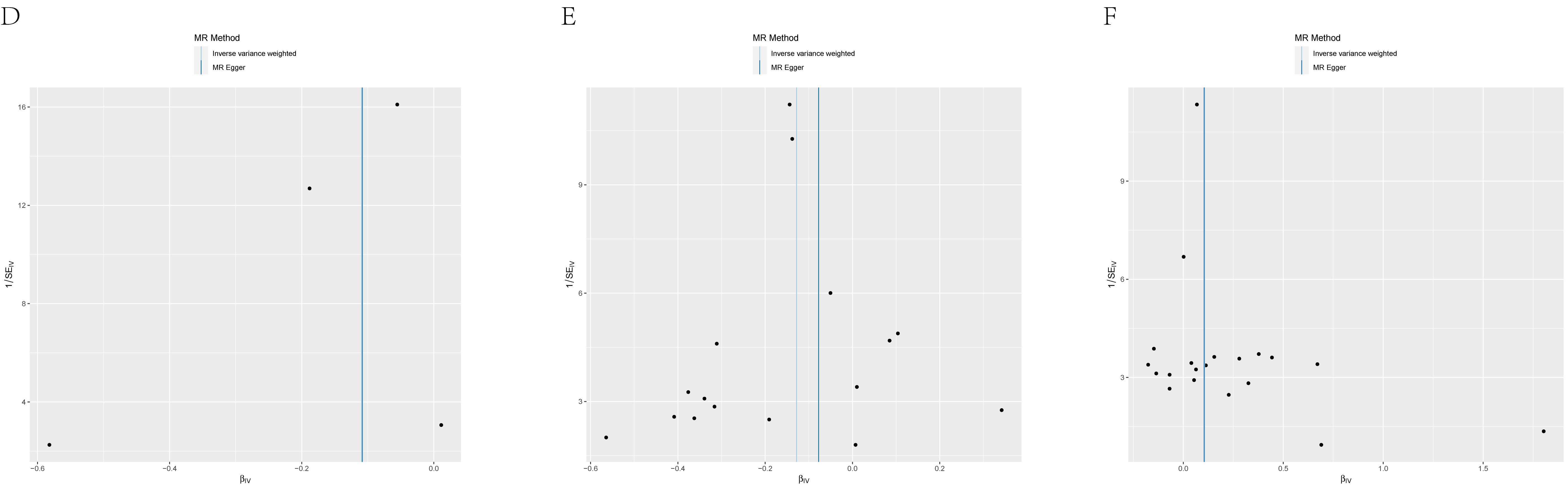


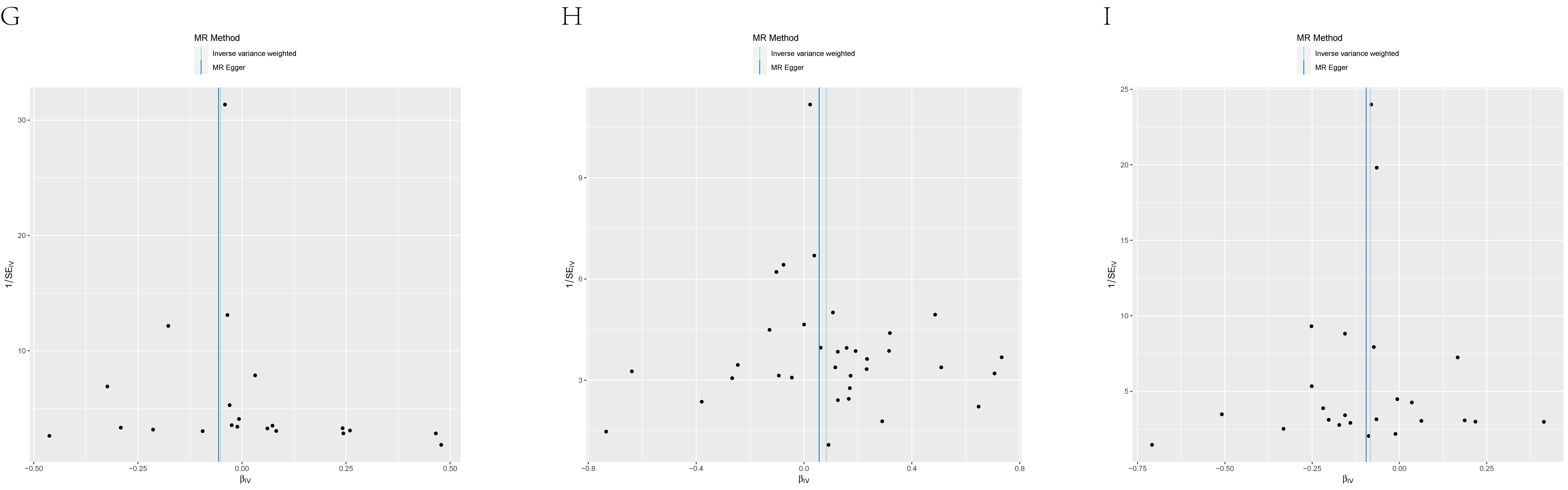


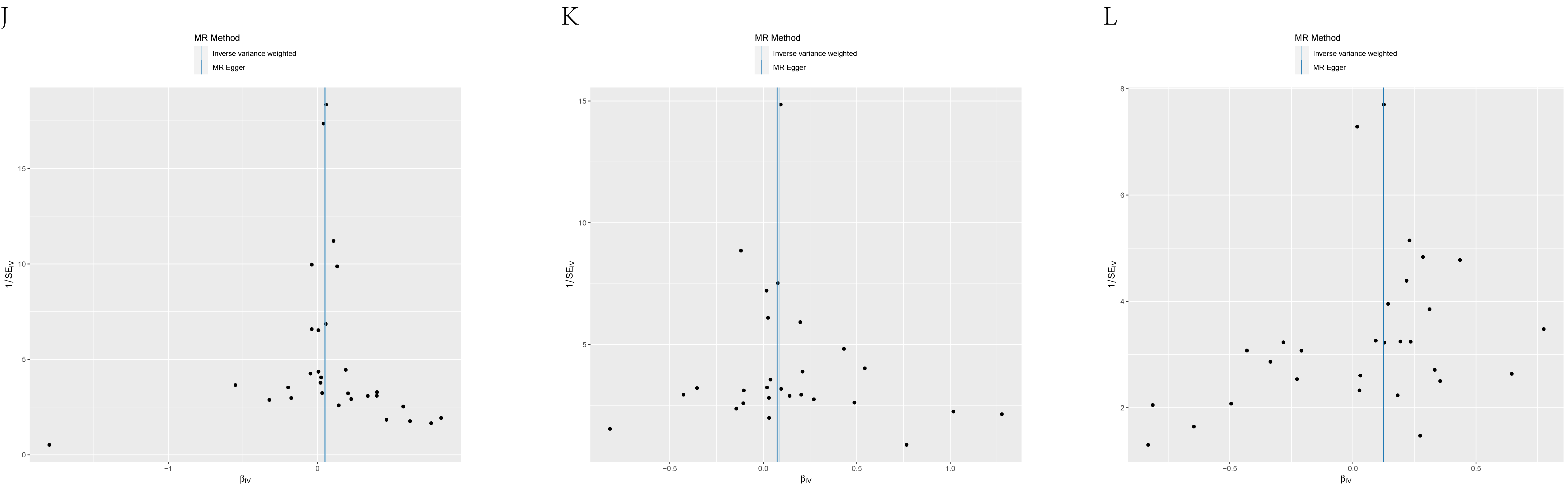


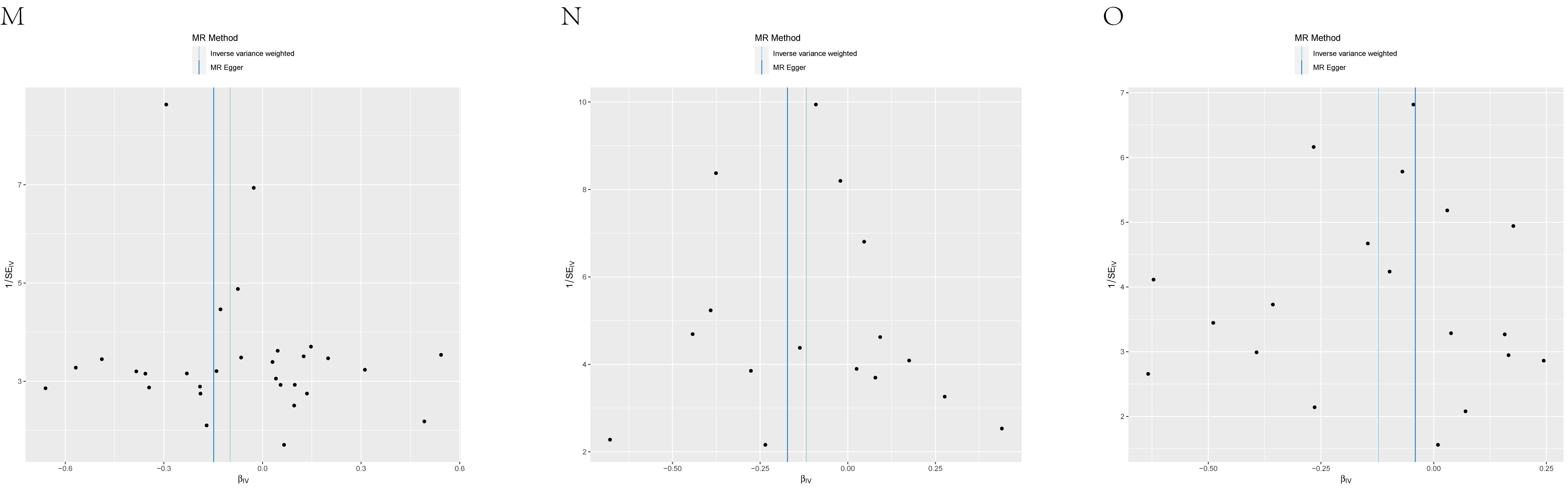


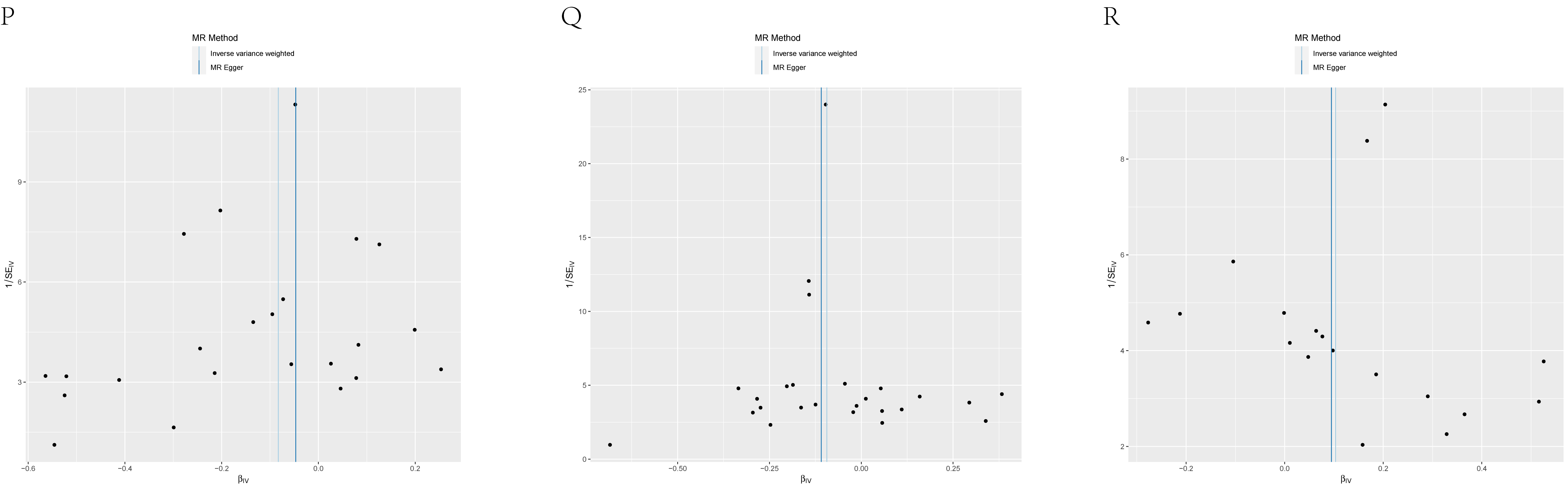


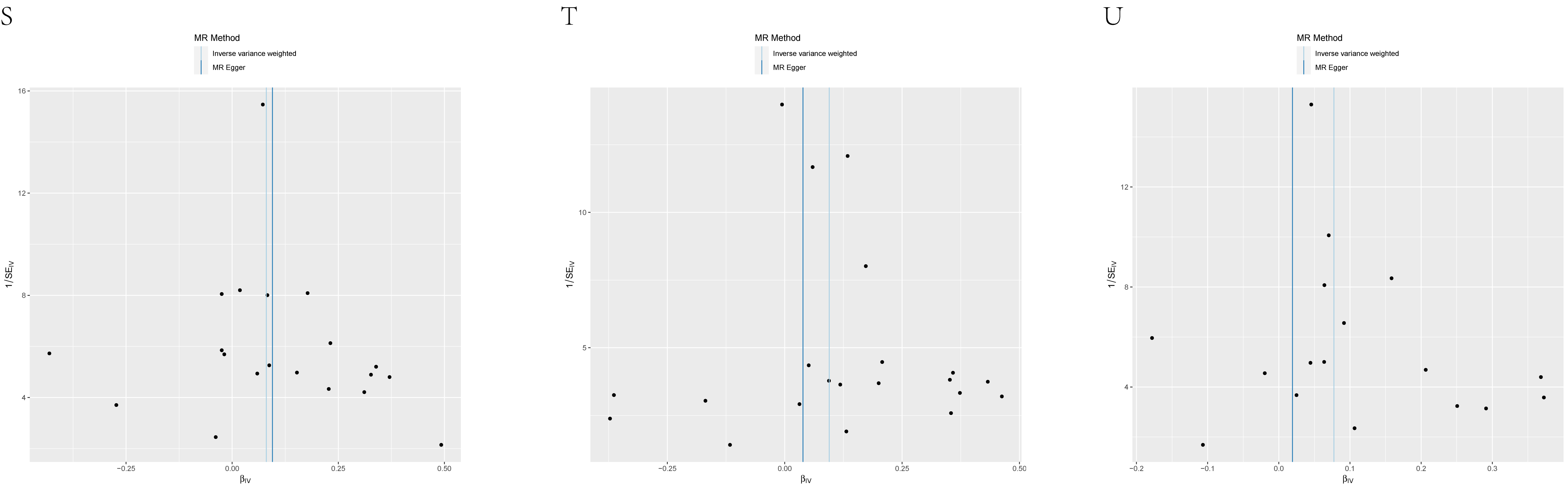


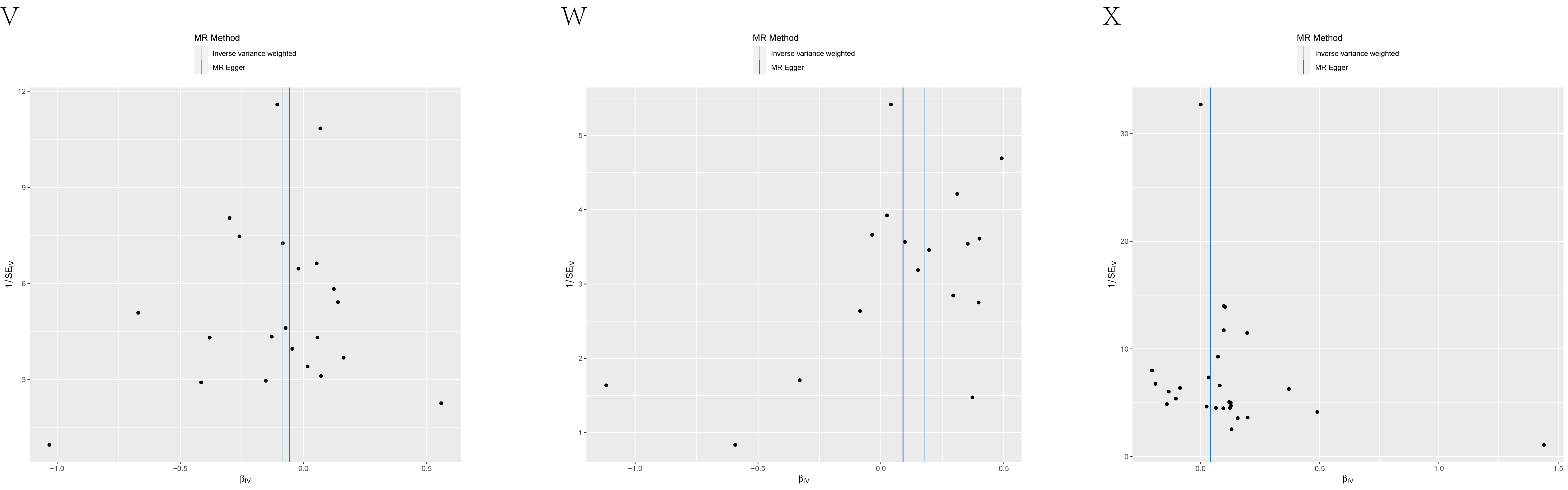


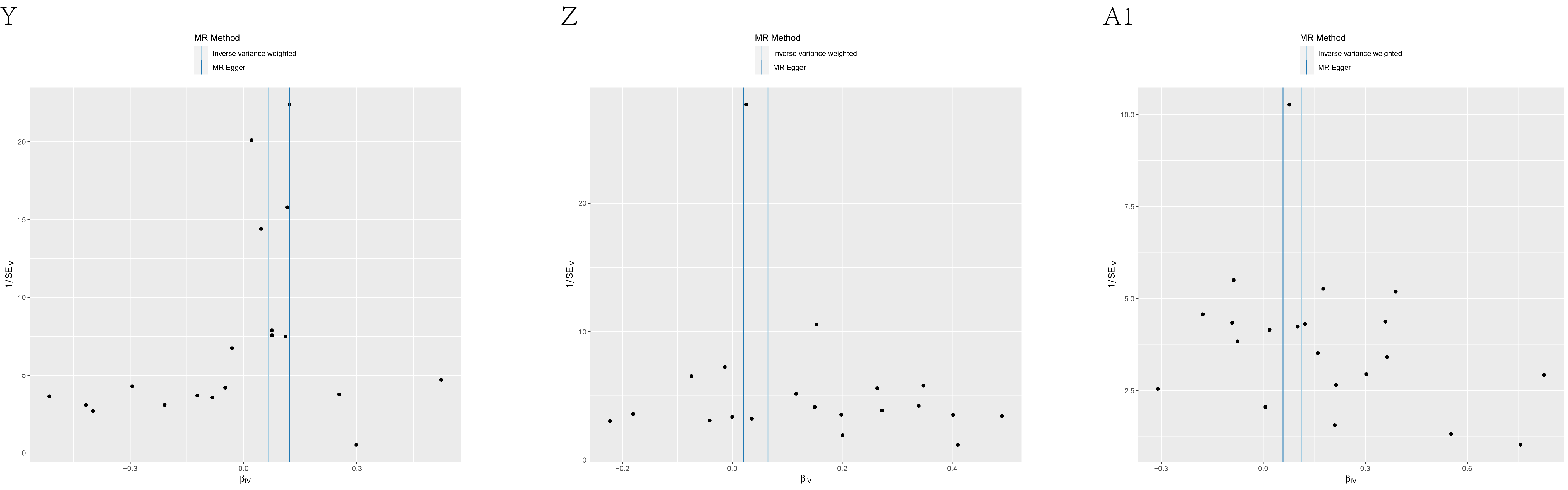


Supplementary Fig. 3 The IVW and MR-Egger tests were employed to evaluate the heterogeneity across 27 immune cell phenotypes linked to pancreatic cancer.

(A) CD11c+ monocyte %monocyte，(B) CD39+ resting Treg %resting Treg，(C) CD4 Treg AC,

(D) CM DN (CD4-CD8-) AC，(E) CM DN (CD4-CD8-) %DN, (F) TD DN (CD4-CD8-) AC,

(G) CD8br AC, (H) NKT AC, (I) HLA DR+ CD4+ AC, (J) HLA DR+ CD8br %T cell,

(K) CD8dim NKT AC, (L) DN (CD4-CD8-) NKT %lymphocyte, (M) B cell AC,

(N)CD20 on IgD+ CD38- naive, (O) CD20 on IgD- CD27-, (P)CD38 on IgD+ CD38dim,

(Q) CD62L on monocyte, (R) CD3 on CD28+ CD45RA- CD8br, (S) HVEM on CD4+,

(T) CD28 on CD39+ secreting Treg, (U) CD28 on CD39+ CD4+, (V) CD45 on NKT,

(W) CD25 on activated Treg, (X) CD33 on CD33br HLA DR+ CD14dim,

(Y) CX3CR1 on CD14- CD16-, (Z) CD39 on CD39+ CD4+, (A1) CD4 on CD28+ CD4+.
