# Supplementary material for: Plasma metabolites as mediators in immune cell-pancreatic cancer risk: insights from Mendelian randomization
Source: Front Immunol. 2024 Jun 12;15:1402113. doi: 10.3389/fimmu.2024.1402113 (PMC11199692; doi:10.3389/fimmu.2024.1402113)
Supplement: Supplementary file 5 [file DataSheet_5.docx]

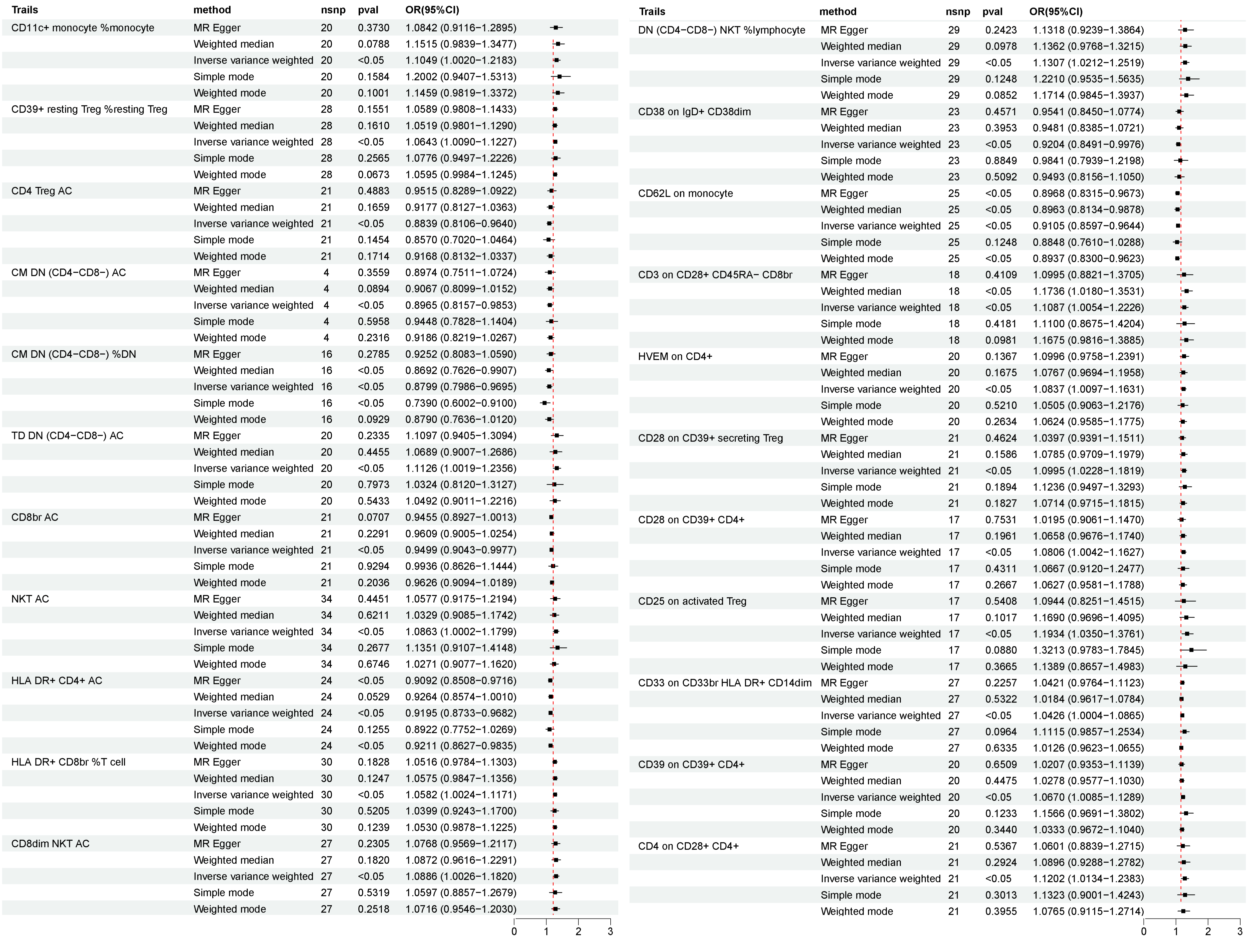


Supplementary Fig. 5 Five different MR analysis methods, consistent directional odds ratios (OR < 1 or OR > 1) were observed for the immune cell phenotypes.
